# Mucocutaneous Manifestations in HIV-Infected Patients and Their Relationship to CD4 Lymphocyte Counts

**DOI:** 10.1155/2020/7503756

**Published:** 2020-08-11

**Authors:** Mina Mirnezami, Nader Zarinfar, Masoomeh Sofian, Bahareh Botlani Yadegar, Hoda Rahimi

**Affiliations:** ^1^Department of Dermatology, Faculty of Medicine, Arak University of Medical Sciences, Arak, Iran; ^2^Department of Infectious Diseases, Arak University of Medical Sciences, Arak, Iran; ^3^Department of Internal Medicine, Isfahan University of Medical Sciences, Isfahan, Iran; ^4^Skin Research Center, Shahid Beheshti University of Medical Sciences, Tehran, Iran

## Abstract

**Background:**

More than 90% of human immunodeficiency virus- (HIV-) infected patients show at least one mucocutaneous manifestation during the course of their disease. The frequency, pattern, and associated factors of these complications vary among different populations.

**Objective:**

This study was planned to evaluate the frequency of cutaneous presentations in HIV-infected patients and their association with the count of CD4 cells.

**Methods:**

A cross-sectional study was conducted on eighty-four HIV-positive patients, who attended the Behavior Consultation Center of Arak University of Medical Sciences. All subjects had a complete physical examination by an expert dermatologist. Further diagnostic procedures were performed, if necessary. Counts of CD4 were determined using flow cytometry.

**Results:**

From 84 patients who enrolled in this study, 95.2% manifested at least one type of mucocutaneous lesions. The most common presentation was xerosis, followed by seborrheic dermatitis, herpes simplex, and oral candidiasis. Oral candidiasis and furuncle were significantly associated with decrease in CD4 cell counts.

**Conclusions:**

Cutaneous manifestations are common in HIV-positive patients, some of which (oral candidiasis and furuncle) could be applicable as useful clinical indicators to predict the immune status of the patients. Therefore, regular skin examinations are recommended as routine HIV-infected patients' healthcare programs.

## 1. Introduction

Human immunodeficiency virus (HIV) involves different tissues and organs, including the skin [1]. Not only are mucocutaneous lesions one of the most common manifestations in acquired immunodeficiency syndrome (AIDS), but also they act as sensitive and useful indicators of the immune status in the patients [1–4].Although these lesions can affect healthy individuals, they are usually more severe, atypical, widespread, and recalcitrant in HIV-infected patients [2]. Skin disorders are classified as primary and secondary in HIV-infected patients. While the pathogenesis of the secondary skin lesions (including opportunistic infections and malignant skin cancers) is associated with decreased CD4 counts, the origin of the primary cutaneous disorders is still being investigated [5]. Despite the significant decrease in the prevalence of opportunistic infections and Kaposi sarcoma at the mercy of using highly active antiretroviral therapy, most cutaneous inflammatory diseases associated with AIDS are still prevalent [5].The primary skin disorders associated with AIDS include seborrheic dermatitis, xerosis, psoriasis, eosinophilic folliculitis, photodermatitis, and cutaneous porphyria [5].

Although skin manifestations are common in HIV patients, their frequency, pattern, and associated factors have been found to vary among different populations. Investigating the prevalence of AIDS-associated skin disorders in different regions of the world is therefore crucial for treating HIV patients. Lowe et al. found skin disorders in 88% of 301 HIV-positive patients, while pruritic skin lesions and plane warts were the most observed ones [6]. Panya et al. reported cutaneous lesions in 85% of 347 HIV-positive patients and noted significant association between pruritic papular eruptions and decreased CD4 counts [7], which was consistent with Noruka's result [1]. Josephine et al. reported generalized pruritus, vaginal candidiasis, and herpes zoster as the most prevalent skin manifestations in 384 HIV-infected patients and found herpes zoster and candidiasis to be significantly associated with low CD4 counts [8].These results were supported by Levy and Jacobson who observed candidiasis in cases with CD4 counts of below 300 [9].In Thailand, Wiwantikit reported single or multiple skin disorders in 80% of 120 HIV-positive patients and found xerosis, candidiasis, seborrheic dermatitis, and pruritic rashes as the most common mucocutaneous presentations [10]. In another study by Kim et al., Kaposi sarcoma was associated with CD4 counts <200, while warts were associated with CD4 counts >200 in HIV-positive patients [11], which was compatible with the results of previous studies [12,13]. Goh et al. found psoriasis and drug reactions to be significantly associated with CD4 counts <200 [14].

## 2. Materials and Methods

This cross-sectional study was conducted on 84 HIV-positive patients, aged over 15 years, presenting to the Behavioral Counseling Center of Arak University of Medical Sciences. The study protocol was approved by the Arak University of Medical Sciences Ethics Committee. All participants were informed of the study protocol, comprehensively, and then they were asked to sign the consent forms. Patients were thoroughly examined by an expert dermatologist. The bacteriological and fungal tests were carried out, and skin biopsies were performed, if necessary. The mucocutaneous manifestations, classified as skin infections (bacterial, fungal, and viral infections), inflammatory lesions, malignancies, and nail disorders, were recorded in a checklist. Blood samples were collected from all patients, and their CD4 cell counts were determined through flow cytometry to assess their immunity status. Statistical analyses were performed using SPSS-16.0 (SPSS Inc., Chicago, Ill., USA). The categorical data were compared using the chi-squared test and the level of statistical significance was set at *P* < 0.05.

## 3. Results

Out of the 84 HIV patients, who aged 23–53 years with a mean age of 34.6 ± 6.3 years, 20 (23.8%) were female and 64 (76.2%) were male. Seventeen patients (20.2%) had CD4 counts < 200, twenty-six (30.8%) had CD4 counts of 200–500, and 41 (49%) had CD4 counts > 500. Sixty-two patients had been infected by the virus through drug abuse, four of which reported a history of suspected sexual contact, as well.

The frequencies of different mucocutaneous manifestations are presented in Table 1. The most common skin disorders were xerosis (54.8%; mean of CD4 count: 460 cell/mm^3^) and seborrheic dermatitis (54.4%; mean of CD4 count: 430 cell/mm^3^). In the infectious category, the most common cutaneous infection was caused by herpes simplex virus (HSV) (52.4%; mean of CD4 count: 405 cell/mm^3^), followed by oral candidiasis (47.6%; mean of CD4 count: 375 cell/mm^3^). The most common hair disorder was telogen effluvium which was observed in 19% of cases. Nail disorders included nail hyperpigmentation (26.2%), nail dystrophy (7.1%), cyanosis and clubbing (4.2%), and onychomycosis (2.4%). Among all studied mucocutaneous lesions, only oral candidiasis (*P*=0.002) and furuncle (*P*=0.006) were significantly associated with low CD4 cell counts.

## 4. Discussion

Although several studies were conducted on skin disorders in HIV-positive patients, the relationship between mucocutaneous lesions and CD4 counts was rarely evaluated. The frequency of mucocutaneous manifestations in HIV-positive patients in different studies was reported as 55.6% [11], 96% [15, 16], and 73.3% [17]. In this study, 95.2% of patients had mucocutaneous lesions, among which the most common ones were xerosis, seborrheic dermatitis, HSV infection, and oral candidiasis. These results were in accordance with the studies reported by Levy et al., Foroughi et al., Josephine et al. and Noruka et al. [1, 8, 9, 18].

The present study found infections with HSV (52.4%) and oral candidiasis (47.6%) to be the most prevalent infectious cutaneous lesions. This finding was compatible with Chopra's study which presented candidiasis as the most common infection, followed by zoster, genital wart, and genital herpes [19]. However, Ali Azfar et al. and his colleagues reported fungal infection as the most common one, followed by viral and bacterial infections [20]. In the present study, no case of skin malignancy and eosinophilic folliculitis was observed which was consistent with the findings of Dwiyana and his colleagues [16]. Conversely, in a study conducted in Tanzania, Kaposi sarcoma was reported one of the most common cutaneous lesions in HIV-positive patients [12].

We found that most of the mucocutaneous infectious lesions were present in patients with CD4 <350, while most of the mucocutaneous noninfectious manifestations were observed in patients with CD4 >350, which were corresponding to the observations of Crum-Cianflone's et al. [13]. The present research also found oral candidiasis to be a mucocutaneous disorder with significant association with low CD4 counts, which is consistent with previous studies [8, 9, 21–24]. Furthermore, a statistically significant association was found between furuncle and low CD4 counts, which was compatible with Uthayakumar's research [25].Nevertheless, Ali Azfar et al. could not show any association between skin manifestations and CD4 cell counts [20]. On the other hand, Fernandes and Bhat reported statistically significant association with CD4 T-cell count in pyodermas, dermatophytoses and papular pruritic eruptions [26]. Lowe et al. reported that seborrheic dermatitis, pruritic popular rashes, and musculum contagiosum were most commonly seen in patients with lower CD4 counts [6]. According to Noruka's research, pruritic papular rashes were most commonly found in CD4 counts <200 and seborrheic dermatitis was observed in patients with CD4 counts between 201 and 500 [1]. In Goh's study, CD4 counts <200 were remarkably associated with psoriasis and drug reactions [14]. However, these correlations were not observed in our study. These discrepancies in the results of different studies could be explained by variations in their sample sizes, stages of the disease, routes of infection, and regional patterns of the reported diseases.

## 5. Conclusion

The present study demonstrated that mucocutaneous manifestations are common in HIV-positive patients, some of which (oral candidiasis and furuncle) could be applicable as useful clinical indicators to predict the immune status of the patients. Therefore, regular skin examinations are recommended as routine HIV-infected patients' healthcare programs. The limitation of our study was its rather small sample size; thus, further multicenter studies with larger sample sizes are recommended to explain the current conflicting results.

## Figures and Tables

**Table 1:** Mucocutaneous manifestations in HIV-infected patients and their relationship with CD4 lymphocyte counts.

Cutaneous manifestation	*N* (%)	CD4 count (*N*)				Mean CD4 count	*P* value
		<200	201–350	351–500	>500		
Pruritic popular eruption	12 (14.3)	0	2	6	4	555	0.2
Oral candidiasis	40 (47.6)	2	20	12	6	375	0.002^*∗*^
Tinea versicolor	4 (4.8)	2	0	0	2	438	0.4
Onychomycosis	2 (2.4)	0	0	0	2	677	0.6
Dermatophytosis	12 (14.3)	2	4	2	4	497	0.9
Zoster	6 (7.1)	0	4	0	2	369	0.8
Herpes simplex	44 (52.4)	6	18	8	12	405	0.2
Chicken pox	2 (2.4)	0	0	2	0	428	0.2
Folliculitis	30 (35.7)	0	10	6	14	502	0.2
Furunculosis	4 (4.8)	4	0	0	0	178	0.006^*∗*^
Impetigo	2 (2.4)	0	2	0	0	192	0.1
Cellulitis	2 (2.4)	2	0	0	0	176	0.1
Eczema	24 (28.6)	4	4	4	12	473	0.8
Seborrheic dermatitis	44 (52.4)	4	18	8	14	430	0.5
Urticaria	4 (4.8)	0	2	0	2	424	0.7
Xerosis	46 (54.8)	8	14	10	14	460	0.7
Hair loss	16 (19)	0	2	4	10	616	0.3
Common wart	4 (4.8)	0	4	0	0	322	0.4
Genital wart	4 (4.8)	0	0	2	2	262	0.2
Acne	12 (14.3)	10	0	2	0	617	0.1
Oral hyperpigmentation	16 (19)	2	6	4	4	369	0.3
Melanonychia	22 (26.2)	6	6	10	0	406	0.1

^*∗*^ *p* value < 0.05.

## Data Availability

Some parts of the data used to support the findings of this study are included within the article (the table) and some part is not freely available due to patients' privacy.
